# Determination of Cutoff Values for DEXA-Based Body Composition Measurements for Determining Metabolic and Cardiovascular Health

**DOI:** 10.1089/biores.2014.0056

**Published:** 2015-01-01

**Authors:** Pierre-Olivier Lang, Christophe Trivalle, Thomas Vogel, Jacques Proust, Jean-Pierre Papazyan, Moustapha Dramé

**Affiliations:** ^1^ Health and Wellbeing Academy, Anglia Ruskin University, Cambridge, United Kingdom.; ^2^ Nescens Centre of Preventive Medicine, Clinic of Genolier, Genolier, Switzerland.; ^3^ Pôle Gériatrie, Hôpitaux Universitaires de Paris-Sud, Hôpital Paul Brousse, Assistance-Publique Hôpitaux de Paris, Villejuif, France.; ^4^ Pôle de Gériatrie, Hôpitaux Universitaires de Strasbourg, Strasbourg, France.; ^5^ Centre of Nuclear Medicine, Clinic of Genolier, Genolier, Switzerland.; ^6^ Department of Research and Innovation, Hôpitaux Universitaires de Reims, Reims, France.; ^7^ Faculty of Medicine, University of Reins-Champagne-Ardenne, Reins, France.

**Keywords:** muscle mass, fat mass muscle mass ratio, fat mass index, metabolic health, cardiovascular diseases

## Abstract

The two components of the body weight (i.e., fat mass and muscle mass) appeared to be of high interest to consider in predicting metabolic health related risks. We aimed to determine cutoff values for fat mass index (FMI) and muscle mass index (MMI), FM/MM, and BMI for metabolic and cardiovascular health. This study was a cross-sectional analysis study conducted in a center of preventive medicine. It included 616 consecutive outpatients: mean age was 56.0±10.0 years (74.6% aged ≥50), and 61.4% were female. Fat and muscle mass were obtained with dual energy X-ray absorptiometry scan analyses. Metabolically unhealthy individuals were defined as people with biological features of dyslipidemia, hyperuricemia, diabetes, and/or hepatitis steatosis. Documented hypertension and/or atherosclerosis of at least one major artery defined individuals with cardiovascular complications. Receiver-operating characteristic curve analysis revealed that the cutoff values for MMI, FMI, and FM/MM were respectively 18.8kg/m^2^ (sensitivity [Se]=58%; specificity [Sp]=59%), 5.5kg/m^2^ (Se=61%; Sp=62%), and 0.31 (Se=62%; Sp=62%) in men; and 14.1kg/m^2^ (Se=52%; Sp=54%), 5.5kg/m^2^ (Se=65%; Sp=67%), 0.39 (Se=73%; Sp=73%) in women for predicting metabolic health. Values were 19.3kg/m^2^ (Se=58%; Sp=59%), 7.0kg/m^2^ (Se=61%; Sp=62%) and 0.49 (Se=62%; Sp=62%) in men; and 15.7kg/m^2^ (Se=58%; Sp=59%), 6.4kg/m^2^ (Se=61%; Sp=62%) and 0.35 (Se=62%; Sp=62%) in women for cardiovascular complications. Whatever the outcomes considered, the Youden indexes for BMI values were systematically below 25 kg/m^2^, except for cardiovascular complications in men, where the threshold for the best Se/Sp was 25.7 kg/m^2^. These cutoff values for FMI, MMI, and FM/MM could be of practical value for the clinical evaluation of a deficit in MM with or without excess of FM. They complement the classical concept of BMI in a more qualitative manner and extend the analysis of its impact on health outcomes to all BMI categories.

## Introduction

Expressed as the relationship between the total body mass in kg and stature in m^2^, the body mass index (BMI) is the most widely used indicator of nutritional status,^1,2^ and its health-related risks.^3^ But limitations in its capacities to account for total adiposity^2,4^ and to predict the development of metabolic and health complications have been highlighted.^5,6^ Furthermore, while fat deposition was usually considered to fit the development of metabolic and health complications, reduced muscle mass (MM) was more recently implicated.^7–12^ Thus, the balance of the two major components of the total body weight have become of interest in predicting nutritional status related health outcomes.^2,4,13^

Indeed, the major shortcomings of BMI is that the composition of body weight is not taken into account: excess body weight may be made up of adipose tissue or conversely muscle hypertrophy, both of which will be judged as “excess mass.”^14^ Inversely, a deficit of BMI may be due to MM deficit (e.g., sarcopenia) or mobilization of fat tissue, or both combined (e.g., starvation). Finally, this two-compartment model of the body weight appears to be of interest to better identify how adult weight may interfere with health outcomes.^4^ Conversely to BMI, population-specific fat-mass and muscle-mass indexes (i.e., FMI and MMI, expressed in kg/m^2^) can be considered for comparative analyses between populations from different ethnicities.^6^ Surprisingly, these indexes have not found a wide application yet, probably because appropriate cutoff values have not been proposed. The present study proposes a cross sectional analysis from a sample of 616 outpatients, to identify cutoff values for FMI, MMI, FM/MM, and BMI for metabolic and cardiovascular complications. All body composition measurements considered were obtained from dual energy X-ray absorptiometry (DEXA) scans analyses.

## Material and Methods

### Study population and design

Patient and data collection have been already published elsewhere.^4^ The sample study was recruited at the Clinic of Genolier (Switzerland). It consisted of 616 consecutive ambulatory patients consulting our center of preventive medicine (www.nescens.com) for a medical checkup between January 1, 2009 and December 31, 2012. Pregnant women and/or individuals with self-reported cardiac failure, who had a cardiac pacemaker, or who had previously undergone limb amputation were not considered. Otherwise, there were not any exclusion criteria.

Briefly, Nescens medical checkups are designed in three steps. The first and third steps are dedicated to medical consultations, which are conducted before and after all the complementary investigations. The medical consultation conducted before is dedicated to personal and family medical histories, medications, current complaints, and symptoms, and a complete medical examination is also performed. In the course of the first consultation, patients were informed about the protocol prior to signing consent forms. The results of the checkup are detailed and discussed during the second consultation. The second step is composed by biological, radiological, and functional investigations. Thus, blood test analyses, including in part the biological data of interest (see below and Table 1 for details), are carried out as well as a large panel of radiology exams such as DEXA scan, angiography by computed tomography scan (CT scan) of the supra-aortic and coronary arteries, and total body CT scan (256-row detector CT scan). In addition, a cardiologist conducts a stress test on electronically braked cycle ergometer in addition to a Doppler echocardiography. In order to avoid any interference due to injection of iodine-based contrast products used for the angiography and total body CT scans, DEXA scans were systematically carried out in first. The data of interest were retrospectively collected from medical files between April 1 and May 31, 2013.

**Table 1.** Characteristics of the Individuals Composing the Sample Study According to Gender

Characteristics		Total *N*=616	Women *N*=378	Men *n*=238	*p-*Value
Age (years)	*mean±SD*	56.0±10.0	55.5±9.7	56.8±10.4	0.02
Age (years), *n* (%)					<0.00001
	20–29	6 (1.0)	6 (1.6)	0 (0.0)	
	30–39	28 (4.6)	8 (2.1)	20 (8.4)	
	40–49	122 (19.8)	88 (23.3)	34 (14.3)	
	50–59	238 (38.6)	152 (40.2)	86 (36.1)	
	60–69	164 (26.6)	90 (23.8)	74 (31.1)	
	≥70	58 (9.4)	34 (9.0)	24 (10.1)	
Country of origin, *n* (%)					<0.00001
	France	68 (11.0)	44 (11.6)	24 (10.1)	
	Russia	78 (12.7)	50 (13.2)	28 (11.8)	
	Switzerland	400 (64.9)	260 (68.8)	140 (58.8)	
	Other^a^	70 (11.4)	24 (6.4)	46 (19.3)	
Personal history of…, *n* (%)					
High blood pressure		116 (18.8)	42 (11.1)	74 (31.1)	<0.00001
Any cardiovascular events		2 (0.3)	0 (0.0)	2 (0.8)	NS
Stroke		2 (0.3)	0 (0.0)	2 (0.8)	NS
Myocardial infarcts		2 (0.3)	0 (0.0)	2 (0.0)	NS
Coronaropathy		38 (6.2)	6 (1.6)	32 (13.4)	<0.00001
Arteriopathy		10 (1.6)	0 (0.0)	10 (4.2)	<0.00001
Any vascular disease,^b^*n* (%)		128 (20.8)	42 (11.1)	86 (36.1)	<0.00001
Risk factor for cardiovascular diseases, *n* (%)					
	Family history	286 (46.4)	166 (43.9)	120 (50.4)	NS
	Tabaco consumption	200 (32.5)	84 (22.2)	116 (48.7)	<0.0001
MUH,^c^*n* (%)		372 (60.4)	208 (55.0)	180 (75.6)	<0.00001
Hepatic steatosis,^d^*n* (%)		214 (34.7)	100 (26.5)	114 (47.9)	<0.00001
Known hypercholesterolemia, *n* (%)		196 (31.8)	88 (23.3)	108 (45.4)	<0.00001
Taking of cholesterol-lowering drugs, *n* (%)		66 (10.7)	28 (7.4)	38 (16.0)	0.001
Diagnosis of diabetes, *n* (%)		24 (3.9)	8 (2.1)	16 (6.7)	0.005
Total cholesterol	mean±SD	5.9±1.1	6.01±1.2	5.7±1.1	0.0008
	≥5.2 mmol/L, *n* (%)	174 (28.3)	130 (34.6)	44 (18.5)	<0.00001
HDL cholesterol	mean±SD	1.7±0.5	1.8±0.5	1.6±0.6	<0.0001
	<0.9 mmol/L, *n* (%)	16 (2.6)	4 (1.1)	12 (5.0)	0.004
Cholesterol/HDL	≥6.5 *n* (%)	22 (3.6)	16 (4.3)	6 (2.5)	NS
LDL cholesterol	mean±SD	3.7±1.0	3.7±1.1	3.7±1.0	NS
	≥3.7 mmol/L, *n* (%)	318 (51.8)	184 (48.9)	134 (56.3)	NS
Triglycerides	mean±SD	1.1±0.6	1.1±0.6	1.3±0.6	<0.0001
	≥1.7 mmol/L, *n* (%)	88 (15.0)	36 (9.6)	52 (21.8)	<0.00001
HbA1c^e^	mean±SD	5.5±0.7	5.4±0.6	5.7±0.9	<0.0001
	>6.5%, *n* (%)	38 (6.23)	20 (5.4)	18 (7.6)	NS
CRP-us^f^	mean±SD	2.0±3.1	1.9±3.1	2.2±3.1	NS
Ferritin^g^	mean±SD	139.8±92.9	119.8±80.6	171.3±101.9	<0.0001
Uric acid^h^	mean±SD	281.4±94.4	255.2±93.9	320.0±80.3	<0.0001
Hyperuricemia		64 (11.3)	46 (13.5)	18 (8.0)	NS
Homocystein^i^	mean±SD	10.5±3.1	10.2±3.2	10.9±2.8	0.006
Lp(a)^j^	mean±SD	253.6±272.6	255.8±261.1	250.9±286.7	NS

*p*<0.05 indicates that the candidate variable is significantly associated with gender; if *p*>0.05, it is not significant (NS).

^a^ From other origin means: Algeria, 4 (0.7%); Belgium, 16 (2.6%); China, 8 (1.3%); Dubai, 2 (0.3%); Egypt, 2 (0.3%); Iraq, 4 (0.7%); Kuwait, 2 (0.3%); Monaco, 6 (1.0%); Saudi Arabic, 24 (3.8%); and Sweden, 2 (0.4%,).

^b^ Any vascular diseases: suffering at least from high blood pressure, any coronaropathy (symptomatic or not), and/or a stenosis of supra aortic trunk (with or without symptoms or complication), and/or an arteriopathy of the lower limbs.

^c^ MUH, metabolically unhealthy, is defined by the presence of at least one metabolic disorder (high total cholesterol, high LDL cholesterol, low HDL cholesterol, high triglycerides, and/or HbA1c>6.5%) and/or a diagnosis of hepatic steatosis.

^d^ Hepatic steatosis diagnosed by ultra-sound of the abdomen and defined as a liver hyperechogenicity.

^e^ HbA1c (haemoglobin glycosylated): normal range for men, 4.0 to 6.0%; normal range for women, 4.0 to 6.0%. Diabetes, if defined when HbA1c value is above 6.5%.

^f^ CRP-us (ultra sensitive C-reactive protein): normal value is<5.0 mg/L.

^g^ Ferritin: normal range for men, 22 to 275 μg/L; normal range for women, 10 to 204 μg/L.

^h^ Uric acid: normal range for men, 210 to 420 μmol/L; normal range for women, 150 to 360 μmol/L.

^i^ Homocystein: normal value<16 μmol/L.

^j^ Lp(a) [lipoprotein (a)]: normal value<300 mg/L.

SD, standard deviation.

### Data collection

#### Assessment of body composition

The assessment of body composition was performed with a DEXA scan (Hologic Discovery; Hologic Inc.).^15^ The instrument was calibrated by using a spine phantom daily and a step phantom weekly. All scans were performed 4–5 h after the last meal, at least, and before CT scans. DEXA represents a three-compartment model for estimating body composition, because it can divide the body into three compartments: fat, bone mineral, and all other fat-free mass that does not include bone. Thus, unlike two-compartment models, DEXA is not subject to errors caused by variations in bone density among different ethnicities. DEXA thus provides bone density estimates, and regional estimates of body composition (i.e., it can estimate the body composition of individual parts of your body), by measuring the body's absorbance of X-rays at two different energies. Fat, bone mineral, and fat-free soft tissue have different absorption properties. DEXA gets estimates of the body composition by scanning the entire body in 5–10 minutes, which were analyzed by a trained technician.

Body-composition component estimates included FM and MM. Subsequent to the measurement of individuals' height, height-adjusted indexes were considered. Thus, height raised to the power of 2 was used to calculate BMI [weight (kg) /height^2^ (m^2^)], FM index [FMI=FM (kg)/ height (m^2^)], and MM index [MMI=MM (kg)/height (m^2^)]. Finally, in order to consider together the two body compartments, the FM/MM was calculated and considered as well.

According to BMI values, for descriptive purposes, individuals were classified as underweight (<18 kg/m^2^), normal weight (18–24.9 kg/m^2^), overweight (25–29.9 kg/m^2^), or obese (≥30 kg/m^2^).^1^ For statistical analyses, 18–24.9 kg/m^2^ was used as reference group because it considers normal weight individuals. Body heights were measured to the nearest 0.5 cm and total body weight to the nearest 0.1 kg with calibrates digital scales (Seca Corp.).

#### Biochemical markers

All biochemical makers considered in the study (see Table 1) were measured at the laboratory of the Clinic of Genolier (Synlab^®^ Suisse, www.synlab.ch), which is accredited according to the international standards (ISO/CEI 15189 STS 497). Fasting blood samples were collected on peripheral venipuncture before all imagery investigations. Serum levels of total cholesterol, high density lipoprotein (HDL) cholesterol, low density lipoprotein (LDL) cholesterol, triglycerides and uric acid were measured using enzymatic methods with Combas Integra^®^ 400 (Hoffman-La Roche Ltd.). Blood glucose was measured with a hexokinase method, and blood HbA1c (glycated hemoglobin) determined by International Federation of Clinical Chemistry and Laboratory Medicine standardized immunoturbidimetric method with Combas Integra^®^ 400. The serum homocystein and lipoprotein Lp(a) levels were measured with nephelometric immunoassays on latex particles using BN ProSpect^®^ (Siemens AG) and Combas Integra^®^ 400 respectively.

### Outcome of interest

#### Metabolic health

Metabolically unhealthy (MUH) individuals were defined as participants who demonstrate biological features of dyslipidemia (i.e., total and/or LDL hypercholesterolemia, HDL hypocholesterolemia, and/or hypertriglyceridemia) and/or hyperuricemia and/or a diagnosis of diabetes defined as a level of HbA1c upper to 6.5%.^16^ A diagnosis of hepatitis steatosis was also part of the definition. The ratio of liver-to-spleen (L/S) Hounsfield units (HU) <1.0 and liver attenuation <40 HU on the abdomen 256-row detector CT scan were used for diagnosing hepatitis steatosis.^17^ The normal serum values for biological markers were those given by the laboratory (www.synlab.ch): total cholesterol <5.2 mmol/L; LDL cholesterol <3.7 mmol/L; HDL cholesterol >0.9 mmol/L; triglycerides <1.7 mmol/L; 210≤uric acid≤420 for men and 150≤uric acid≤360 for women; and 4.0≤HbA1c≤6.0 for both genders.

#### Cardiovascular diseases

Medical data and diagnostics of interest leading to identify individuals suffering from metabolic-related cardiovascular complications were collected throughout the two medical consultations from previous reports, personal medical history, and the result of the tests performed during the checkup. Thus, individuals who demonstrate documented hypertension (under treatment or not) and/or a significant coronaropathy (symptomatic or not), atherosclerosis of at least one of the supra-aortic arteries (symptomatic or not) and/or of one large arteries of the body were considered as having cardiovascular diseases.^16^

### Statistical analyses

For descriptive analysis, numerical variables are presented as mean±standard deviation (m±SD) and for categorical variables as number and percentage. Statistical tests used for the unifactorial comparative analysis were chosen according to the type of variable, the sample size under consideration, and the number of group compared. Thus, numerical outcomes were compared using analysis of variance or Kruskal-Wallis test (when >2 groups), and Student's *t* or Mann-Whitney test (when 2 groups). For categorical outcomes, chi-squared or Fisher's exact test was used. All these analyses were performed with SAS^®^ software (version 9.3; SAS System, SAS Institute Inc., Cary, NC). The level of significance was set at *p*=0.05.

In addition, receiver-operating characteristic (ROC) curves were computed using SPSS^®^ (version 21; SPSS Inc., Chicago IL). Each point on the ROC curve represents a sensitivity/100-specificity pair corresponding to a particular decision threshold. The choice for cutoff values was that value on the ROC curve move from the vicinity of the upper left corner over toward the lower right corner (i.e., Youden index); the accuracy was the area under the ROC curve (AUROC). ROC curve analyses were computed for each body-composition component estimates (MM, MMI, FM, FMI, and FM/MM) and BMI values, this in the whole analytic sample and according to gender. The significance level was set at 0.05 and corresponds to the probability that the observed sample AUROC is found. If *p*<0.05, then AUROC is significantly different from 0.5.

## Results

In total, 616 adults composed the analytic sample. Their characteristics are summarized in Table 1 and anthropometric and DEXA scan parameters in Table 2. Mean age was 56.0 (SD 10.0) years; 61.4% were females. Respectively, 73% and 77% of women and men were aged 50 years or over. One-fifth of the sample presented at least one cardiovascular complications; their prevalence was significantly higher in men. While women mostly presented high blood pressure (11.1%), men demonstrated high blood pressure (31.1%), but also coronaropathy (13.4%) or an arteriopathy of one of the other main body arteries (4.2%). Biologically, 60.4% of the analytic sample was metabolically unhealthy (MUH). Whether there was no significant difference between genders for serum LDL cholesterol levels, all other parameters were significantly more prevalent in men.

**Table 2.** Results of the Descriptive Analysis of the Anthropometric and DEXA-Based Data According to Gender

Anthropometric data	Total *N*=616	Women *N*=378	Men *n*=238	*p-*Value
Height (cm)	169.7±9.5	165.2±7.7	176.9±7.5	<0.0001
Total body weight (kg)	68.6±18.1	60.5±15.1	81.4±14.7	<0.0001
Body mass index (kg/m^2^)	23.7±5.5	22.3±5.9	25.9±3.8	<0.0001
Body mass index categories, *n* (%):				<0.0001
BMI<18 kg/m^2^	114 (18.5)	106 (28.1)	2 (3.4)	
18≤BMI<24.9 kg/m^2^	282 (45.8)	188 (49.7)	94 (39.5)	
25≤BMI<29.9 kg/m^2^	118 (19.2)	40 (10.6)	78 (32.8)	
BMI≥30 kg/m^2^	102 (16.5)	44 (11.6)	58 (24.3)	
**DEXA scan body composition**				
Muscle mass (kg)	47.1±12.2	39.9±7.3	58.5±9.2	<0.0001
Muscle mass index (kg/m^2^)	16.2±3.1	14.6±2.6	18.6±2.2	<0.0001
Percentage of muscle mass	69.2±8.0	67.1±7.2	72.6±8.0	<0.0001
Fat mass (kg)	19.1±9.2	18.4±9.2	20.2±4.9	0.003
Fat mass index (kg/m^2^)	6.7±3.4	6.8±3.7	6.5±2.9	NS
Percentage of fat mass	27.2±8.4	29.2±7.9	24.1±8.3	<0.0001
Fat mass/muscle mass	0.40±0.2	0.45±0.2	0.32±0.2	<0.0001

All data given as mean±SD unless otherwise noted.

BMI, body mass index.

Obviously all anthropometric data and DEXA scan parameters were higher in men compared with women, except FMI (*p*>0.05) and the percentage of FM, which was higher in women (*p*<0.0001) (Table 2). According to BMI, while women were mostly classified into the normal (49.7%) and underweight (28.1%) categories, the vast majority of men were uniformly distributed within normal (39.5%), overweight (32.8%) and obese (24.3%) groups.

**FIG. 1.**
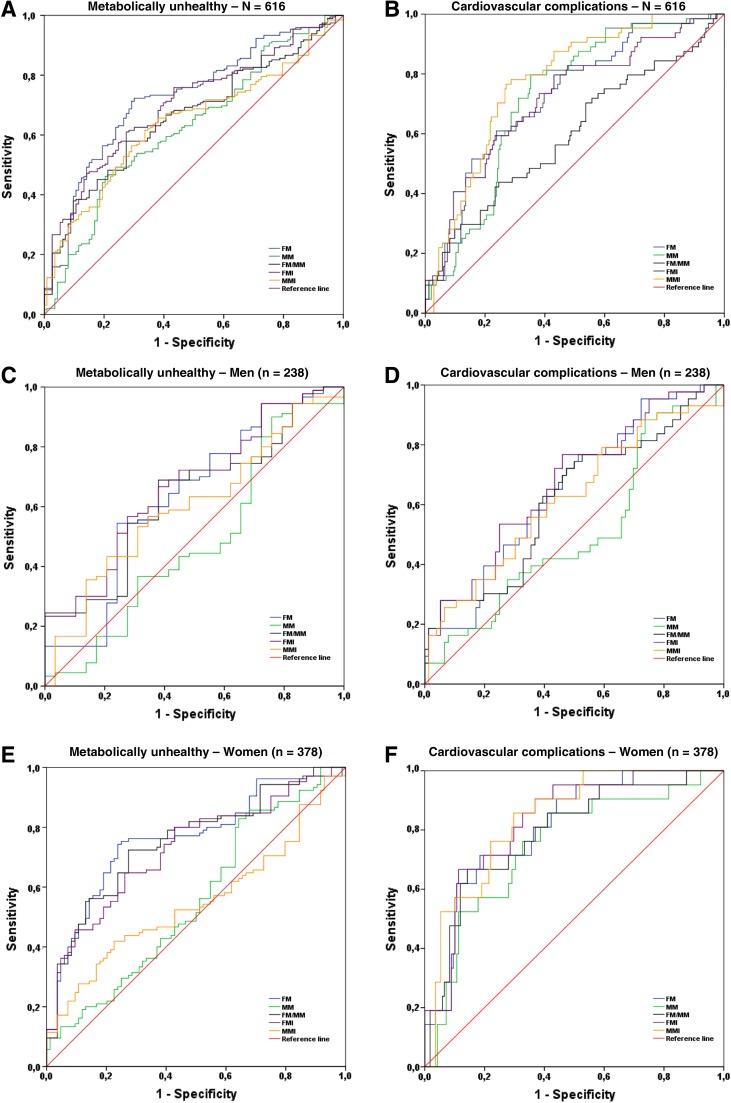
Receiver operating characteristic (ROC) curves for DEXA scan based measurements of the body composition in predicting metabolically unhealthy status and cardiovascular complications in the analytic sample (panels *A* and *B*), and according to gender (for men see panels *C* and *D*, and for women see panels *E* and *F*) respectively. MM, muscle-mass; MMI, muscle mass index; FM, fat mass; FMI, fat mass index; FT/MM, fat mass to muscle mass ratio.

ROC curves analysis is presented in Figure 1. The comparisons of the capability of the FM, FMI, MM, MMI, FM/MM, and BMI to predict metabolic and cardiovascular health are presented in Tables 3 and 4 respectively. The highest accuracy was systematically measured in women compared with men, and for the prediction of cardiovascular complications compared with metabolic health. FMI systematically showed the highest area under curve compared with MMI and FM/MM. MM related parameters had the lowest capability to predict the outcomes and the AUROC were even insignificantly different from 0.5 for the prediction of the metabolic health. ROC curve analysis revealed that the optimal cutoff values for MMI, FMI, and FM/MM are respectively 18.8 kg/m^2^ (sensitivity [Se]=58%; specificity [Sp]=59%), 5.5 kg/m^2^ (Se=61%; Sp=62%), and 0.31 (Se=62%; Sp=62%) in men; and 14.1 kg/m^2^ (Se=52%; Sp=54%), 5.5 kg/m^2^ (Se=65%; Sp=67%), and 0.39 (Se=73%; Sp=73%) in women for predicting metabolic health; values are 19.3 kg/m^2^ (Se=58%; Sp=59%), 7.0 kg/m^2^ (Se=61%; Sp=62%), and 0.49 (Se=62%; Sp=62%) in men; and 15.7 kg/m^2^ (Se=58%; Sp=59%), 6.4 kg/m^2^ (Se=61%; Sp=62%), and 0.35 (Se=62%; Sp=62%) in women for cardiovascular complications. For all these cutoff values, the Sp was systematically better than the Se, and these parameters were better for the prediction of cardiovascular health than metabolic one.

**Table 3.** Comparison of the Capability of the Different DEXA Scan Based Body Components and Body Mass Index to Predict the Metabolically Unhealthy Status in the Whole Analytic Sample and According to Gender

Total (*N*=616)	Youden index	Sensitivity (%)	Specificity (%)	Accuracy	95% CI	*p*-Value
Muscle mass (kg)	42.3 kg	59	60	0.626	0.580–0.671	0.02
Muscle mass index (kg/m^2^)	15.5 kg/m^2^	65	62	0.640	0.597–0.684	<0.0001
Fat mass (kg)	15.5 kg	71	71	0.725	0.684–0.766	<0.0001
Fat mass index (kg/m^2^)	5.3 kg/m^2^	65	62	0.702	0.660–0.743	<0.0001
Fat mass/muscle mass	0.4	64	61	0.660	0.617–0.703	0.02
BMI (kg/m^2^)	21.7 kg/m^2^	69	35	0.694	0.653–0.736	<0.0001
**Women** (*n*=378)						
Muscle mass (kg)	38.3 kg	50	50	0.555	0.496–0.614	NS
Muscle mass index (kg/m^2^)	14.1 kg/m^2^	52	54	0.549	0.491–0.607	NS
Fat mass (kg)	13.8 kg	76	77	0.761	0.713–0.810	<0.0001
Fat mass index (kg/m^2^)	5.5 kg/m^2^	65	67	0.729	0.697–0.779	0.02
Fat mass/muscle mass	0.39	73	73	0.753	0.704–0.801	<0.0001
BMI (kg/m^2^)	19.4 kg/m^2^	62	50	0.612	0.555–0.670	<0.0001
**Men** (*n*=238)						
Muscle mass (kg)	60.1 kg	45	45	0.483	0.392–0.574	NS
Muscle mass index (kg/m^2^)	18.8 kg/m^2^	58	59	0.600	0.519–0.681	0.02
Fat mass (kg)	16.8 kg	60	62	0.634	0.547–0.721	0.002
Fat mass index (kg/m^2^)	5.4 kg/m^2^	61	62	0.669	0.591–0.746	<0.0001
Fat mass/muscle mass	0.31	62	62	0.633	0.553–0.712	0.002
BMI (kg/m^2^)	24.0 kg/m^2^	70	45	0.703	0.638–0.769	<0.0001

Youden index is the cutpoint value, which is the value on the receiver-operating characteristic (ROC) curve that will move from the vicinity of the upper left corner over toward the lower right corner.

Sensitivity measures the proportion of actual positives, which are correctly identified as such (e.g., the percentage of individuals who are correctly identified as having the condition=the true positive rate).

Specificity measures the proportion of negatives which are correctly identified as such (e.g., healthy people who are correctly identified as not having the condition=the true negative rate).

Accuracy represents the area under the ROC curve (AUROC), which is the measure of how well the parameter discriminate healthy and unhealthy status.

95% confidence interval.

*p-*Value<0.05 indicates that the AUROC is significantly different from 0.5 that corresponds to the vicinity of the lower left corner toward the upper right corner; NS=nonsignificant, meaning *p*≥0.05.

**Table 4.** Comparison of the Capability of the Different DEXA Scan Based Body Components and BMI to Predict Cardiovascular Complications in the Whole Analytic Sample and According to Gender

Total (*N*=616)	Youden index	Sensitivity (%)	Specificity (%)	Accuracy	95% CI	*p*-Value
Muscle mass (kg)	47.0 kg	72	69	0.720	0.676–0.764	<0.0001
Muscle mass index (kg/m^2^)	17.2 kg/m^2^	75	73	0.775	0.734–0.817	<0.0001
Fat mass (kg)	17.1 kg	70	61	0.725	0.677–0.772	<0.0001
Fat mass index (kg/m^2^)	6.6 kg/m^2^	66	66	0.717	0.666–0.768	<0.0001
Fat mass/muscle mass	0.40	56	57	0.597	0.538–0.655	0.001
BMI (kg/m^2^)	23.8 kg/m^2^	81	32	0.814	0.780–0.848	<0.0001
**Women** (*n*=378)						
Muscle-mass (kg)	40.8 kg	71	70	0.744	0.665–0.823	<0.0001
Muscle-mass index (kg/m^2^)	15.7 kg/m^2^	76	78	0.836	0.783–0.890	<0.0001
Fat-mass (kg)	17.5 kg	72	74	0.808	0.747–0.870	<0.0001
Fat-mass index (kg/m^2^)	7.0 kg/m^2^	72	72	0.829	0.772–0.887	<0.0001
Fat-mass/muscle-mass	0.49	72	72	0.789	0.717–0.862	<0.0001
BMI (kg/m^2^)	22.6 kg/m^2^	85	27	0.855	0.810–0.900	<0.0001
**Men** (*n*=238)						
Muscle mass (kg)	58.8 kg	45	45	0.506	0.428–0.583	NS
Muscle mass index (kg/m^2^)	19.3 kg/m^2^	60	60	0.669	0.599–0.740	<0.0001
Fat mass (kg)	19.2 kg	60	60	0.648	0.578–0.719	<0.0001
Fat mass index (kg/m^2^)	6.4 kg/m^2^	60	60	0.619	0.543–0.695	0.002
Fat mass/muscle mass	0.35	60	60	0.616	0.541–0.692	0.03
BMI (kg/m^2^)	25.7 kg/m^2^	68	45	0.703	0.638–0.769	<0.0001

Youden index is the cutpoint value, which is the value on the receiver-operating characteristic (ROC) curve that will move from the vicinity of the upper left corner over toward the lower right corner.

Sensitivity measures the proportion of actual positives, which are correctly identified as such (e.g., the percentage of individuals who are correctly identified as having the condition=the true positive rate).

Specificity measures the proportion of negatives which are correctly identified as such (e.g., healthy people who are correctly identified as not having the condition=the true negative rate).

Accuracy represents the area under the ROC curve (AUROC), which is the measure of how well the parameter discriminate healthy and unhealthy status.

95% confidence interval.

*p*-Value<0.05 indicates that the AUROC is significantly different from 0.5 that corresponds to the vicinity of the lower left corner toward the upper right corner; NS=nonsignificant, meaning *p*≥0.05.

As presented in Tables 3 and 4, optimal cutoff values for BMI are 24.0 kg/m^2^ (Se=70%; Sp=45%) and 19.4 kg/m^2^ (Se=62%; Sp=50%) in men and women respectively for predicting metabolic health. For cardiovascular complications, a similar trend is observed with cutoff values of 22.6 kg/m^2^ (Se=85%; Sp=27%) in women and 25.7 kg/m^2^ (Se=68%; Sp=45%) for men. Except the last, all these cutoff values are within the normal weight category according to the World Health Organization obesity classification.

## Discussion

The main finding of this study is that FM/MM is at least as sensitive and specific as FMI and MMI considered alone in predicting metabolic and cardiovascular health in adults of both gender (Tables 3 and 4). But FM/MM considers simultaneously the impact of both components on health outcomes. Furthermore, in this analytic sample, cutoff values measured for BMI were not in overweight or obese category but within the normal weight category according to the World Health Organization obesity classification,^4^ when the two health outcomes were considered.

However, while ROC analysis provides a useful means to assess the diagnostic accuracy of a test and to compare the performance of more than one test for the same outcome, this usefulness must to be considered in the light of the clinical circumstances.^18^ Indeed, the Se and Sp may not be invariant for a test, but may depend on characteristics of the population. Thus, we have analyzed the capability to predict metabolic and cardiovascular health in 616 outpatients and according to gender. In men and women, for the both outcomes considered the Se (62% vs. 72%) and Sp (62% vs. 72%) were similar for FM/MM. While women were more classified into under- and normal-weight categories (77.8%) according to BMI classification; men were in majority (57.1%) within the overweight and obese categories (Table 2). The higher prevalence of metabolic unhealthy status and cardiovascular complications in men (75% and 36.1%) compared with women (55% and 11.1%), also contributed to lower the accuracy, Se and Sp in men. By calculating the AUROC and given to the *p* value and 95% confidence interval, FMI and FM/MM significantly help to predict the two health outcomes and help to discriminate healthy from unhealthy individuals.^18^

Cutoff values for FM/MM were systematically higher for cardiovascular complications (men=0.35; women=0.49) than for metabolic health (men=0.31; women=0.39). This can be explained by the natural course of the metabolic disease. Indeed, fatty deposition as well as reduction in MM are associated with insulin-resistance and elevated serum concentration of C-reactive protein, interleukin 6 or 8, and tumor necrosis factor-α^1^ that progressively induce metabolic disorders which ultimately turn to cardiovascular complications.^13^ In their turn, insulin resistance and low-grade chronic inflammation both favor abdominal fatty deposition^19^ and increase protein catabolism.^20^ While biologically this perpetuates the proinflammatory status, it also contributes to intensify the unbalance between the two body components and hence increases the FM/MM.

Thus, partitioning total body weight in the sum of FM and MM is of particular interest because not only increased adiposity,^1^ but also reduced MM (*via* the inhibition of insulin signaling transduction^11^ and disruption in skeletal muscle mitochondria^12^) are both associated with the development of major metabolic and cardiovascular complications. Conversely, reduced adiposity and maintenance of a good MM are protective^7–9^ Although we believe that the definition of obesity based on relative fat (i.e., percentage of fat) remains of great value, it is important to mention that a person can lose weight without subsequently changing his/her fat mass (i.e., in the case of crash diets). This interest of dissecting the body weight is furthermore highlighted when you consider that in the present study the cutoff values for BMI are far from the values usually considered as associated with metabolic and health risks.^3^

The partitioning of body weight into fat and muscle component is however not possible without assessment of body composition. With this aim, dual energy X-ray absorptiometry (DEXA) is becoming a widespread tool of assessment as well as bioelectrical impedance equations. These two techniques are less costly and more accessible than the gold standard imaging methods, computed tomography (CT scan) and magnetic resonance imaging^15^ and are now well-validated and reliable technique to measure the different body components.^21^ Compared with the bioelectrical impedance equation, however, DEXA does not seem to produce any bias.^22^ In addition, although it is much more expensive test compared with usual anthropometric measurements, in different population, DEXA-based body composition parameters have demonstrated to be more informative for the detection of unhealthy conditions and metabolic risk factors than total body weight, BMI, waist circumference, waist-to-hip ratio.^15^ Thus, DEXA has become a validated, reliable, and safe technique to assess the body composition. Indeed, in terms of possible radiation exposure problems, this technics uses the equivalent of less than 10% of one day's exposure to natural background radiation (0.001 mSv), which corresponds to lower level of radiation than a standard X-ray (0.1 mSv).^4,21^ In addition, it is important to remind that DEXA was initially developed to explore the bone density. Interestingly, it has thus been well demonstrated that all biological features of metabolic syndrome adversely impacts bone metabolism in men and women as well,^23,24^ and the body composition in its whole contributes to bone health.^25,26^

Finally, it is well accepted that the positive association between body mass and bone mass density does not make obesity and osteoporosis mutually exclusive.^26^ Thus, DEXA scan may provide simultaneously information about the three body weight components.^4,15^ Furthermore, there is mounting evidence that the distribution and type of excess fat (subcutaneous fat vs. visceral fat cells) is an important prognostic indicator for disease risk.^27,28^ Even subjects who are normal weight and have a BMI <25 kg/m^2^ can have a significant accumulation of visceral fat.^1,4^ Recent advancement in DEXA scan technologies makes this method able to differentiate visceral from subcutaneous fat. This method of measurement is highly correlated and linearly related to visceral fat measurements by computed tomography.^29^ Thus, analyzing body composition components, including visceral fat, through DEXA would allow clinicians and researchers to classify subjects with excess visceral fat and/or muscle mass deficit, thereby identifying the population with the greatest risks for major metabolic and health complications and subsequently to design personalized interventions to maximize health benefits. But while DEXA is the most reliable technique to estimate body composition (i.e., excess of fat mass, muscle mass deficit, and bone density)^21^ it has source of error. However, DEXA measurements in the present study were made on the same machine (model Discovery W, Hologic Inc.), which is the one used for the National Health and Nutrition Examination Survey body composition reference data.^30^ If it is believed that software upgrades may change the algorithm that the device uses to calculate body composition, the upgrades performed during the study period have always been made to update the same mode of functioning, and multiple controls have certified the reproducibility of the measurements.

Another source of error with the use of DEXA scan is that it relies on the relationship between body composition and body water content. Indeed, this may be disturbed in pathological states that increase whole body water, such as acute cardiac or renal failure. While this point is more particularly a concern when trying to measure change over time, individuals with heart failure were not considered in our analytic sample, and in individuals with chronic renal disease, MM mass would be overestimated.^8^

## Conclusion

The present study has provided cutoff values for FMI, MMI, and FM/MM that could be of practical value for the clinical evaluation of nutritional status. They complement the classical concept of BMI in a more qualitative manner and above all extend the analysis of its impact on health outcomes to all BMI categories. Thus, these cutoff values are attractive parameters for future study of metabolic disorders, not only to estimate the nutritional status and to better identify how body weight may interfere with our health, but also to improve the prevention of metabolic syndrome and its impact on health outcomes. However, further investigations that include FMI, MMI, and FM/MM are necessary. On the basis of longitudinal studies and in the larger population, the interest in and performance of these DEXA-based indices to predict metabolic and cardiovascular outcomes as well as prolonged and healthier longevity should be compared with classical anthropometric measurements.

## Abbreviations Used

AUROC: area under the ROC curve
BMI: body mass index
CT: computed tomography
DEXA: dual energy X-ray absorptiometry
HDL: high density lipoprotein
LDL: low density lipoprotein
FM: fat mass
FMI: fat mass index
OR: odds ratio
MUH: metabolically unhealthy
MHO: metabolically healthy overweight
MUHO: metabolically unhealthy overweight
MM: muscle mass
MMI: muscle mass index
ROC: receiver-operating characteristic

## References

[1] Lang PO, Mahmoudi R, Novella JL, et al. Is obesity a marker of robustness in vulnerable hospitalized aged population? Prospective, multicenter cohort study of 1306 Acutely ill patients. J Nutr Health Aging. 2014;18:66–74 24402392 10.1007/s12603-013-0352-9 PMC12878474

[2] Heymsfield SB, Cefalu WT Does body mass index adequately convey a patient's mortality risk? JAMA. 2013;309:87–88 23280230 10.1001/jama.2012.185445

[3] Flegal KM, Kit BK, Orpana H, Graubard BI Association of all-cause mortality with overweight and obesity using standard body mass index categories: a systematic review and meta-analysis. JAMA. 2013;309:71–82 23280227 10.1001/jama.2012.113905 PMC4855514

[4] Lang PO, Trivalle C, Vogel T, Proust J, Papazian JP Markers of metabolic and cardiovascular health in adults: Comparative analysis of DEXA-based body composition components and BMI categories. J Cardiol. 2014;4 29 pii: [Epub ahead of print]; DOI: 10.1016/j.jjcc.2014.03.010 24794756

[5] Deurenberg P, Deurenberg-Yap M, Guricci S Asians are different from Caucasians and from each other in their body mass index/body fat per cent relationship. Obes Rev. 2002;3:141–146 12164465 10.1046/j.1467-789x.2002.00065.x

[6] Dhesi JK, Bearne LM, Moniz C, et al. Neuromuscular and psychomotor function in elderly subjects who fall and the relationship with vitamin D status. J Bone Miner Res. 2002;17:891–897 12009020 10.1359/jbmr.2002.17.5.891

[7] Lee S, Kim Y, White DA, Kuk JL, Arslanian S Relationships between insulin sensitivity, skeletal muscle mass and muscle quality in obese adolescent boys. Eur J Clin Nutr. 2012;66:1366–1368 23073260 10.1038/ejcn.2012.142 PMC3656505

[8] Srikanthan P, Karlamangla AS Relative muscle mass is inversely associated with insulin resistance and prediabetes. Findings from the Third National Health and Nutrition Examination Survey. J Clin Endocrinol Metab. 2011;96:2898–2903 21778224 10.1210/jc.2011-0435

[9] Pagel-Langenickel I, Bao J, Pang L, Sack MN The role of mitochondria in the pathophysiology of skeletal muscle insulin resistance. Endocrine Rev. 2010;31:25–51 19861693 10.1210/er.2009-0003 PMC2852205

[10] Atlantis E, Martin SA, Haren MT, Taylor AW, Wittert GA Inverse associations between muscle mass, strength, and the metabolic syndrome. Metabolism. 2009;58:1013–1022 19394973 10.1016/j.metabol.2009.02.027

[11] Wolfe RR The underappreciated role of muscle in health and disease. Am J Clin Nutr. 2006;84:475–482 16960159 10.1093/ajcn/84.3.475

[12] Lowell BB, Shulman GI Mitochondrial dysfunction and type 2 diabetes. Science. 2005;307:384–387 15662004 10.1126/science.1104343

[13] Alam I, Ng TP, Larbi A Does inflammation determine whether obesity is metabolically healthy or unhealthy? The aging perspective. Mediators Inflamm. 2012;2012:456456 23091306 10.1155/2012/456456 PMC3471463

[14] Schutz Y, Kyle U, Pichard C Fat-free mass index and fat mass index percentiles in caucasians aged 18–98 y. Int J Obesity. 2002;26:953–960 10.1038/sj.ijo.0802037 12080449

[15] Taylor AE, Kuper H, Varma RD, et al. Validation of dual energy X-ray absorptiometry measures of abdominal fat by comparison with magnetic resonance imaging in an Indian population. PLoS One. 2012;7:e51042 23272086 10.1371/journal.pone.0051042 PMC3522679

[16] Wildman RP, Muntner P, Reynolds K, et al. The obese without cardiometabolic risk factor clustering and the normal weight with cardiometabolic risk factor clustering: prevalence and correlates of 2 phenotypes among the US population (NHANES 1999–2004). Arch Intern Med. 2008;168:1617–1624 18695075 10.1001/archinte.168.15.1617

[17] Speliotes EK, Massaro JM, Hoffmann U, et al. Liver fat is reproducibly measured using computed tomography in the Framingham Heart Study. J Gastroenterol Hepatol. 2008;23:894–899 18565021 10.1111/j.1440-1746.2008.05420.x PMC3057524

[18] Bewick V, Cheek L, Ball J Statistics review 13: receiver operating characteristic curves. Critical Care. 2004;8:508–512 15566624 10.1186/cc3000 PMC1065080

[19] Krentz AJ, Viljoen, Sinclar A Insulin resistance: a risk marker for disease and disability. Diabet Med. 2012;30:535–548 23173973 10.1111/dme.12063

[20] Lang PO, Michel JP, Zekry D Frailty syndrome: a transitional state in a dynamic process. Gerontology. 2009;55:539–549 19346741 10.1159/000211949

[21] Sun G, Cahill F, Gulliver W, T et al. Concordance of BAI and BMI with DXA in the Newfoundland population. Obesity (Silver Spring). 2013;21:499–503 23404962 10.1002/oby.20009

[22] Nigam P, Misra A, Colles SL Comparison of DEXA-derived body fat measurement to two race-specific bioelectrical impedance equations in healthy Indians. Diabetes Metab Syndr. 2013;7:72–77 23680244 10.1016/j.dsx.2013.02.031

[23] Zhou J, Zhang Q, Yuan X, et al. Association between metabolic syndrome and osteoporosis: a meta-analysis. Bone. 2013;57:30–35 23871747 10.1016/j.bone.2013.07.013

[24] Hwang DK, Choi HJ The relationship between low bone mass and metabolic syndrome in Korean women. Osteoporos Int. 2010;21:425–431 19565174 10.1007/s00198-009-0990-2

[25] Marin RV, Pedrosa MA, Moreira-Pfrimer LD, Matsudo SM, Lazaretti-Castro M Association between lean mass and handgrip strength with bone mineral density in physically active postmenopausal women. J Clin Densitom. 2010;13:96–101 20171571 10.1016/j.jocd.2009.12.001

[26] Dytfeld J, Ignaszak-Szczepaniak M, Gowin E, Michalak M, Horst-Sikorska W Influence of lean and fat mass on bone mineral density (BMD) in postmenopausal women with osteoporosis. Arch Gerontol Geriatr. 2011;53:e237–242 21281972 10.1016/j.archger.2011.01.002

[27] Bergman RN, Kim SP, Catalano KJ, Hsu IR, Chiu JD, Kabir M et al. Why Visceral Fat Is Bad: Mechanisms of the Metabolic Syndrome. Obesity. 2006;14:16S–19S 16642958 10.1038/oby.2006.277

[28] Weltman A, Despres JP, Clasey JL, et al. Impact of abdominal visceral fat, growth hormone, fitness, and insulin on lipids and lipoproteins in older adults. Metabolism. 2003;52:73–80 12524665 10.1053/meta.2003.50007

[29] Micklesfield LK, Goedecke JH, Punyanitya M, Wilson KE, Kelly TL Dual-energy x-ray performs as well as clinical computed tomography for the measurement of visceral fat. Obesity. 2012;20:1109–1114 22240726 10.1038/oby.2011.367 PMC3343346

[30] Kelly TL, Wilson KE, Heymsfield SB Dual energy X-Ray absorptionmetry body composition reference values. PLoS One. 2009;4:e7038 19753111 10.1371/journal.pone.0007038 PMC2737140

